# The association between physical activity and symptoms of depression in different contexts – a cross-sectional study of Norwegian adolescents

**DOI:** 10.1186/s12889-018-6257-0

**Published:** 2018-12-12

**Authors:** Annette Løvheim Kleppang, Ingeborg Hartz, Miranda Thurston, Curt Hagquist

**Affiliations:** 1 Faculty of Social and Health Sciences, Department of Public Health, Inland Norway University of Applied Sciences, Campus Elverum, Terningen Arena, PO Box 400, 2418 Elverum, Norway; 2 Centre for Research on Child and Adolescent Mental Health, Karlstad University, Karlstad, Sweden; 3 Division of Epidemiology, Norwegian Institute of Public Health, Oslo, Norway; 4 Inland Hospital Trust, Oppland, Norway

**Keywords:** Adolescent, Physical activity, Informal group and team sport context, Symptoms of depression

## Abstract

**Background:**

The purpose was to analyse the association between physical activity taking place in different contexts (sports club, gym, exercise independently and other organized physical activities) and symptoms of depression.

**Methods:**

The study was based on self-reported cross-sectional data from the Ungdata survey, conducted in 2017 by the Norwegian Social Research (NOVA) institute in cooperation with regional centres for drug rehabilitation. The target group comprised 5531 15–16 years old adolescents (Grade 10 students) and 11,655 students in grades 8 and 9 in Norway. Based on Rasch analysis, six items on depressions symptoms were used to create a composite measure of depression. Binomial logistic regression was used to analyse the association between physical activities in different contexts and symptoms of depression.

**Results:**

In the crude model, the odds for symptoms of depression were lower for those who were physically active in a sports club (OR: 0.40, 95% CI: 0.30–0.53), in a sports club and gym (OR: 0.40, 95% CI: 0.28–0.56), in a sports club and exercise or keep fit independently (OR: 0.52, 95% CI: 0.38–0.72) and in a sports club, gym and exercise or keep fit independently (OR: 0.58, 95% CI: 0.41–0.81). After adjustment for potential confounders, the association became significant only for those who were physically active in a sports club (OR: 0.57, 95% CI: 0.40–0.81) and other organized physical activities, e.g. dance, martial arts (OR: 0.52, 95% CI: 0.31–0.86). Analysis for grade 8 and 9 showed the same patterns for the associations between sports club and symptoms of depression (grade 8: crude model, OR: 0.47, adjusted model, OR: 0.63, grade 9: crude model, OR: 0.44, adjusted model, OR: 0.49).

**Conclusions:**

Physical activity in a sports club was associated with significantly lower odds for symptoms of depression, suggesting a possible role for social interaction in addition to physical activity per se. It is important, therefore, to consider in which types of contexts physical activities take place, not only focusing on physical activity frequency and volume when investigating the association between adolescents’ physical activity and mental health. Additional research is needed to further explore these associations as well as measures of physical activity and mental health.

## Background

Mental health problems among youth, including depressive symptoms, have been identified as an important public health issue in Norway in common with most Western countries [1, 2]. Identifying factors in adolescents that can reduce the risk of mental health problems is therefore important if efforts to prevent their occurrence are to be effective [3, 4]. While research on the role of physical activity and cardiovascular health is extensive, interest in the putative role of physical activity on mental health has only more recently emerged. In 2011, however, Biddle & Asare [5] (p. 10) concluded that “Physical activity has potentially beneficial effects for reduced depression, but the evidence base is limited.” Although the research base has expanded since then, the results present a rather mixed picture. A European cross-sectional study, for example, found no evidence of the benefit of daily physical activity for mental health in adolescents [6] and earlier studies [7, 8] showed only a weak association between physical activity and mental health outcomes. However, a recent longitudinal study found that US adolescents who engaged in higher frequencies of physical activity were more resilient to developing depressive symptoms [9].

However, the methodological quality of many studies among adolescents has been questioned; not only are studies primarily of a cross-sectional design but different measurements of mental health and physical activity have been used. Furthermore, if physical activity is conceptualised as a complex multidimensional social practice then the different forms of physical activity and the contexts within which they take place might be important for understanding its relationship with specific mental health outcomes. Factors such as psychological climate (“The experience of the physical activity and the context within which physical activity takes place”) and the characteristics of social interactions, for example, have rarely been accounted for [5]. Exercising or competing in a sports club or keeping fit independently (running, swimming, cycling, walking), may represent different ‘physical activity modalities’ with varying social mechanisms which give rise to different qualitative experiences. According to this conceptualisation, any outcomes are hypothesized to relate to the nature of the experience. Eime et al. [10] have proposed a model based on this type of reasoning but conclude that the model needs further research.

Research efforts have focused on the team sport context when investigating social interaction [11] as well as the organised sport context led by a coach [12]. Brunet et al. [13] reported that involvement in team sport during adolescence and early adulthood is associated with lower symptoms of depression, however, these associations were no longer significant after controlling for covariates (sex, age and parental level of education). Sports participation has been found to be associated with reduced levels of depression and stress among adolescents [14]. Similarly, young people (aged 17–37 years) who participated in team sports (UK university sports people) reported higher levels of happiness than those who played individual sports [15]. A longitudinal study among Canadian adolescents reported that being part of a sports team is associated with lower depressive symptoms [16], which is consistent with a cross-sectional study among German adolescents [17]. A cross-sectional study among European adolescents reported that the lowest levels of depression and anxiety were among those participating in team sports compared with individual sport participation [6]. However, for both sexes, participating in any sport (team or individual) was associated with more positive mental health, independently of frequency of activity. A study among American collegiate athletes showed that the prevalence of mental health conditions was lower for athletes participating in a team sport than in an individual sport [18]. Taken together, these findings suggest that social factors may be important in understanding the mechanism through which physical activity might be related to the prevention of depressive symptoms and highlight the need to differentiate between the different types of physical activity modalities that are assessed in future research [13]. By social factors, we are referring to the presence of other people in the environment, which provide opportunities for interactions. This is especially relevant given that adolescents report an increase in non-organized physical activity and a decrease in organized sport as they move through adolescence [19, 20].

To address this gap, we examined physical activity participation in different contexts to identify the types of physical activity (which include various sports) that might be associated with symptoms of depression.

## Methods

This study was based on data retrieved from the Ungdata surveys conducted by the Norwegian Social Research (NOVA) institute in cooperation with regional centres for drug rehabilitation (KoRus). Ungdata is a repeated cross-sectional study, designed for local surveys of adolescents in Norway. It is financed by the Norwegian Directorate of Health, The Ministry of Children, Equality and Social Inclusion, and the Ministry of Justice and Public Security. Ungdata started in 2010, and, since then, repeated cross-sectional surveys have been conducted in many secondary schools all over the country. The Ungdata survey covers different aspects of young people’s lives, i.e., leisure time activities, health issues, relationships with friends and parents, local environment, school issues, and symptoms of depression. The Ungdata survey has become an important source of information on adolescents’ health and wellbeing, both at the municipal and national level. In this study, we included adolescents from secondary schools, grade 10, aged 15–16 years (and grade 8 and 9) from 19 municipalities in four counties (belonging to Korus east: Hedmark, Oppland, Østfold and Akershus) conducting the Ungdata Survey in 2017. The web-based questionnaire was administered anonymously at school during a school hour with a teacher present. Adolescents were informed that participation was voluntary, and parents were informed in advance through the school learning portal. Altogether 5531 adolescents (grade 10), and 11,655 students in grade 8 and 9 participated. The response rate in the secondary schools we included in the study (for grades 8–10), varied between municipalities (54.2 - 92%).

Table 1 gives the Ungdata Survey 2017 questions, response alternatives and variable definitions included in this study.

**Table 1:** Ungdata Survey 2017: questions, response alternatives and variable definitions

Questions	Response alternatives	Variable definitions
Symptoms of depression		
During the past week, have you been affected by any of the following issues:		
Felt that everything is a struggle (item 1)	Not been affected at all, not been affected much, been affected quite a lot and been affected a great deal.	Symptoms of depression > 80th percentiler
Had sleep problems (item 2)		
Felt unhappy, sad or depressed (item 3)		
Felt hopelessness about the future (item 4)		
Felt stiff or tense (item 5)		
Worried too much about things (item 6)		
Physical activity		
How often do you do physical activity, which gets you out of breath or makes you sweaty?	Never, rarely, 1–2 times a month, 1–2 times a week, 3–4 times a week and at least 5 times a week.	less-than or equal to 2 times a week, > 2 times a week
How often do you participate in the following activities?		
Train or compete with a sports club		
Go to the gym		
Exercise or keep fit independently (running, swimming, cycling, walking)		
Other kinds of organized physical activity (dance, martial arts, etc.)		
Gender		
Are you a boy or a girl?	Boy, girl	
Smoking		
Do you smoke?	I’ve never smoked, I used to smoke but I’ve stopped completely now, I smoke less than once a week, I smoke every week but not every day and I smoke everyday.	No smoking, smoking
Alcohol use		
Do you ever drink any kinds of alcoholic drinks?	I’ve just tried tasting them a few times, occasionally but less than once a month, generally 1–3 times a month and every week.	< 1 time a month
		> 1–3 times a month
Parents higher education		
Did your father and mother go to university or to a university college? Select one answer for each parent. If you are not in touch with one or both of your parents, the skip the question about that parent.	Yes, no	Both parents, One of the parents, None of the pa rents
Family economy		
Financially, has your family been well off, or badly off, over the past years?	We have been well off the whole time, we have generally been well off, we have neither been well off nor badly off, we have generally been badly off, we have been badly off the whole time	Good economy, nor bad or good economy, bad economy

## Measures

### Symptoms of depression

Symptoms of depression were measured with six items derived from a depressive symptoms scale which was derived from the Hopkins Symptom Checklist - 90 [21, 22]. The adolescents were asked if during the past week they have been affected by any of the following: “Felt that everything is a struggle (item 1)”, “had sleep problems (item 2)”, “felt unhappy, sad or depressed (item 3)”, “felt hopelessness about the future (item 4)”, “felt stiff or tense (item 5)”, “worried too much about things (item 6)”. The six questions have four response categories: “Not been affected at all (1)”, “not been affected much (2)”, “been affected quite a lot (3)” and “been affected a great deal (4)”.

Rasch Measurement Theory [23, 24] was used to examine the psychometric properties of the depressive symptoms scale. The scale shows good reliability (Person Separation Index: 0.81). At a general level of analysis, the items work relatively well except for item 2, ‘Sleeping difficulties’, which clearly misfit. The DIF-analysis using ANOVA based on standardized residual of each person to each item indicated evidence of DIF for some items. According to the F-values calculated in the ANOVA, item 6 about “worried too much about things” shows the greatest magnitude of gender-DIF. Given the same location on the latent variable girls score higher than boys on this item. After that item was split into two separate items, one for boys and one for girls, no item showed significant DIF given an adjusted sample size of 540 persons. The Person Separation Index was about the same for both item sets (0.81 vs 0.80). A comparison of the person mean values for the original set of 6 items and the item set resolved for gender-DIF in item 6 showed that the gender differences decreased after resolving the DIF. In order to examine if, and to what extent, the distortion of the outcome variable due to DIF would have an impact on the regression analyses, tentative sensitivity analyses were carried out. The general pattern showing the highest odds ratios for sports club activity remained also when a dependent variable based on an item set with item 6 resolved for gender DIF was used. However, the associations became a bit stronger for girls and a bit weaker for boys. Also, among boys previously non-significant odds ratios for gym and other organized physical activity became significant.

While there are some weaknesses and clearly room for improvements, as a whole the depression scale works reasonable well.

In the Rasch analysis the non-linear raw scores were transformed to person estimates on a linear interval logit scale on which each person is allocated a location (logit) value. These person estimates were used in the statistical analysis in the present study. Lower values on the scale indicate a lower degree of depressive symptoms.

For the purpose of this study, the depression scale was dichotomized based on the latent depression variable generated by the Rasch analysis. A cut off point, reported as logit value, was set at the 80th percentile (0.551), implying two categories on the scale: a) depressive symptoms (≥80th percentile) and b) no depressive symptoms (<80th percentile).

### Physical activity

The participants were asked how often they did physical activity which got them out of breath or made them sweaty, categorized as never, rarely, 1–2 times a month, 1–2 times a week, 3–4 times a week, at least 5 times a week.

### Different types of physical activity contexts

The participants were asked how often they participated in the following activities, categorized as: exercising or competing with a sports club, going to the gym, exercising or keeping fit independently (running, swimming, cycling, walking), other kinds of organized physical activity (dance, martial arts, etc.). These items have been used in the Young in Norway study [25], and have been included in the Ung data survey since 2010. The term “sports club”, refers to training or competing in a sports club, individually or in a team (organized). The term “go to the gym”, refers to exercising/training on their own or together with friends (unorganized). The term “other kinds of organized PA (dance, martial arts, etc.)”, refers to other PA activities (not sports club) that are organized. “Train/exercise or keep fit independently”, refers to unorganized exercising (running, swimming, cycling, and walking etc. without a coach).

Response alternatives included never, rarely, 1–2 times a month, 1–2 time a week, 3–4 times a week and at least 5 times a week. Participation was dichotomized into > 2 times a week and ≤ 2 times a week.

A new variable was created to separate each possible physical activity (PA) context, also including all existing combinations of activities. Physical activities performed less than 3 times a week were classified as non-regular (category 16 below). This was done to examine if the adolescents were physically active in more than one physical activity context, and to investigate the adolescents who only participated in a sports club, or gym, or independently, or in other organized physical activities. The new variable consisted of 16 different possibilities, adolescents participating in: a sports club (1), a sports club and gym (2), a sports club and other organized PA (3), a sports club and independently (4), a sports club, gym and other organized PA (5), sports club, gym and independently (6), sports club, independently and other organized PA (7), sports club, gym, independently and other organized PA (8), gym (9), gym and other organized PA (10), gym and independently (11), gym, independently and other organized PA (12), other kinds of organized PA (dance, martial arts etc.) (13), independently and other organized PA (14), independently (running, swimming, cycling etc. (15) and no regular exercise (16).

Risk factors for many common mental disorders have been shown to be associated with socioeconomic status (SES) [26]. Gender and SES have been included in our analysis to ensure adjustment for well-known confounders to the main association studied in this paper (physical activity contexts and symptoms of depression).

### Parent’s level of education and family economy

Parents’ level of education was measured separately for each parent by asking the following: Did your father/mother go to university or to a university college? Those young people not in touch with one or both parents, were asked to miss the question out. This was categorized as yes or no. Parental educational status was stratified as “no university education”, “one parent with university education” or “both parents have with university education”. Family economy were measured as follows: financially, has your family been well off, or badly off, over the past years? This was categorized as: we have been well off the whole time, we have generally been well off, we have neither been well off nor badly off, we have generally been badly off, we have been badly off the whole time. Family economy was stratified as good, neither bad nor good, and bad economy.

### Smoking and alcohol use

Smoking was measured by asking: do you smoke? And categorized as: I’ve never smoked, I used to smoke but I’ve stopped completely now, I smoke less than once a week, I smoke every week but not every day and I smoke every day. Smoking was dichotomized as “no smoking” and “smoking”. Alcohol consumption was measured by asking: do you ever drink any kinds of alcoholic drinks? This was categorized as never, I’ve just tried tasting them a few times, occasionally, but less than once a month, generally 1–3 times a month, and every week. Alcohol use was dichotomized as “< 1 time a month” and “> 1 - 3 times a month”.

### Analysis

Descriptive contingency table and logistic regression analyses were conducted using SPSS 24.0 for Windows. In the descriptive analyses, the study population was stratified according to symptoms of depression and gender. (Baseline characteristics were presented as proportions with 95% confidence intervals (CI) in each stratum. No overlap of the CI was considered significant at the 5% level).

The Rasch analysis was performed using the software RUMM2030 [27].

Binomial logistic regression analysis was performed to examine the association between different types of physical activity contexts and depressive symptoms, adjusted for confounding variables. A *p*-value of ≤0.05 was set as the level for statistical significance. Associations were presented as odds ratios (OR) with 95% CI, and with adjustments for gender, parent’s education, family economy, smoking and alcohol consumption. The analysis (model A-F) was repeated with adolescents from grades 8 and 9, in order to examine the pattern of the relationship from grade 8 to 10 (results not reported in tables but available on request).

## Results

Table 2 shows baseline characteristics of the study population, aged 15–16 years in 2017, according to depressive symptoms and gender.

**Table 2:** Youth data surveys: baseline characteristics of adolescents, aged 15–16 years in 2017, according to depression symptoms and gender

	Total (*N* = 5531)		Boys (*N* = 2485)		Girls (N = 2485)	
	No depressive symptoms (*N* = 4230)	Depressive symptoms (*N* = 994)	No depressive symptoms (*N* = 2230)	Depressive symptoms (*N* = 255)	No depressive symptoms (*N* = 1795)	Depressive symptoms (*N* = 690)
Physical activity						
How often do you do physical activity, which gets you out of breath or makes you sweaty? (*N* = 5224)						
Never	57 (1.3; 1.0–1.7)	28 (2.8; 1.8–3.9)	29 (1.3; 0.8–1.8)	8 (3.1; 1.0–5.3)	28 (1.6; 1.0–2.1)	19 (2.8; 1.5–4.0)
Rarely	250 (5.9; 5.2–6.6)	87 (8.8; 7.0–10.5)	119 (5.3; 4.4–6.3)	23 (9.0; 5.5–12.5)	116 (6.5; 5.3–7.6)	61 (8.8; 6.7–11.0)
1–2 times a month	231 (5.5; 4.8–6.2)	70 (7.0; 5.5–8.6)	106 (4.8; 3.9–5.6)	18 (7.1; 3.9–10.2)	120 (6.7; 5.5–7.8)	50 (7.2; 5.3–9.2)
1–2 times a week	1047 (24.8; 24.5–26.1)	274 (27.6; 24.8–30.3)	507 (22.7; 21.0–24.5)	56 (22.0; 16.9–27.09	488 (27.2; 25.1–29.2)	204 (29.6; 26.2–33.0)
3–4 times a week	1491 (35.2–33.8-36.7)	305 (30.7; 27.8–33.6)	764 (34.3; 32.3–36.2)	78 (30.6; 24.9–36.2)	661 (36.8; 34.6–39.1)	211 (30.6; 27.1–34.0)
At least 5 times a week	1154 (27.3; 25.9–28.6)	230 (23.1; 20.5–25.8)	705 (31.6; 29.7–33.5)	72 (28.2; 22.7–33.8)	382 (21.3; 19.4–23.2)	145 (21.0; 18.0–24.1)
How often do you participate in the following activities?						
Train or compete with a sports club (*N* = 5202)						
Never	1844 (43.8; 42.1–45.1)	560 (56.3; 53.3–59.4)	892 (40.0; 38.0–42.0)	131 (51.4; 45.2–57.5)	871 (48.5; 46.2–50.8)	404 (58.6; 54.9–62.2)
Rarely	260 (6.1; 5.4–6.9)	62 (6.2; 4.7–7.7)	142 (6.4; 5.4–7.4)	14 (5.5; 2.7–8.3)	103 (5.7; 4.7–6.8)	44 (6.4; 4.6–8.2)
1–2 times a month	102 (2.4; 2.0–2.9)	21 (2.1; 1.2–3.0)	51 (2.3; 1.7–2.9)	5 (2.0; 0.3–3.7)	46 (2.6; 1.8–3.3)	14 (2.0; 1.0–3.1)
1–2 times a week	445 (10.5; 9.6–11.4)	85 (8.6; 6.8–10.3)	236 (10.6; 9.3–11.9)	30 (11.8; 7.8–15.7)	188 (10.5; 9.1–11.9)	52 (7.5; 5.6–9.5)
3–4 times a week	899 (21.3; 20.0–22.5)	152 (15.3; 13.1–17.5)	509 (22.8; 21.1–24.6)	44 (17.3; 12.6–21.9)	346 (19.3; 17.5–21.1)	102 (14.8; 12.1–17.4)
At least 5 times a week	662 (15.7; 14.6–16.8)	110 (11.1; 9.1–13.0)	391 (17.5; 16.0–19.1)	28 (11.0; 7.1–14.8)	221 (12.3; 10.8–13.8)	70 (10.1; 7.9–12.4)
Go to the gym *(N = 5184)*						
Never	1648 (39.0; 37.5–40.4)	404 (40.6; 37.6–43.7)	819 (36.7; 34.7–38.7)	110 (43.1; 37.1–49.2)	764 (42.6; 40.3–44.9)	273 (39.6; 35.9–43.2)
Rarely	594 (14.0; 13.0–15.1)	109 (11.0; 9.0–12.9)	309 (13.9; 12.4–15.3)	22 (8.6; 5.2–12.1)	257 (14.3; 12.7–15.9)	83 (12.0; 9.6–14.5)
1–2 times a month	231 (5.5; 4.8–6.2)	63 (6.3; 4.8–7.9)	98 (4.4; 3.5–5.3)	12 (4.7; 2.1–7.3)	114 (6.4; 5.2–7.5)	47 (6.8; 4.9–8.7)
1–2 times a week	787 (18.6; 17.4–19.8)	186 (18.7; 16.3–21.1)	403 (18.1; 16.5–19.7)	31 (12.2; 8.2–16.2)	329 (18.3; 16.5–20.1)	145 (21.0; 18.0–24.1)
3–4 times a week	669 (15.8; 14.7–16.9)	168 (16.9; 14.6–19.2)	390 (17.5; 15.9–19.1)	55 (21.6; 16.5–26.6)	250 (13.9; 12.3–15.5)	104 (15.1; 12.4–17.7)
At least 5 times a week	264 (6.2; 5.5–7.0)	61 (6.1; 4.6–7.6)	180 (8.1; 6.9–9.2)	22 (8.6; 5.2–12.1)	65 (3.6; 2.8–4.5)	35 (5.1; 3.4–6.7)
Exercise or keep fit independently (running, swimming, cycling, walking) *(N = 5198)*						
Never	859 (20.3; 19.1–21.5)	230 (23.1; 20.5–25.8)	542 (24.3; 22.5–26.1)	81 (31.8; 26.1–37.5)	272 (15.2; 13.5–16.8)	137 (19.9; 16.9–22.8)
Rarely	827 (19.6; 18.4–20.8)	172 (17.3; 15.0–19.7)	438 (19.6; 18.0–21.3)	33 (12.9; 8.8–17.1)	356 (19.8; 18.0–21.7)	130 (18.8; 15.9–21.8)
1–2 times a month	796 (18.8; 17.6–20-0)	171 (17.2; 14.9–19.6)	350 (15.7; 14.2–17.2)	37 (14.5; 10.2–18.8)	403 (22.5; 20.5–24.4)	131 (19.0; 16.1–21.9)
1–2 times a week	957 (22.6; 21.4; 23.9)	204 (20.5; 18.0–23.0)	470 (21.1; 19.4–22.8)	48 (18.8; 14.0–23.6)	442 (24.6; 22.6–26.6)	143 (20.7; 17.7–23.8)
3–4 times a week	473 (11.2; 10.2–12.1)	119 (12.0; 10.0–14.0)	256 (11.5; 10.2–12.8)	27 (10.6; 6.8–14.4)	189 (10.5; 9.1–12.0)	86 (12.5; 10.0–14.9)
At least 5 times a week	300 (7.1; 6.3–7.9)	90 (9.1; 7.3–10.8)	156 (7.0; 5.9–8.1)	23 (9.0; 5.5–12.5))	123 (6.9; 5.7–8.0)	59 (8.6; 6.5–10.6)
Other kinds of organized physical activity (dance, martial arts, etc.) *(N = 5138)*						
Never	2957 (70.0; 68.5–71.3)	670 (67.4; 64.5–70.2)	1667 (74.8; 73.0–76.6)	193 (75.7; 70.4–81.0)	1134 (63.2; 60.9–65.4)	446 (64.6; 61.2–68.2)
Rarely	320 (7.6; 6.8–8.4)	69 (6.9; 5.4–8.5)	167 (7.5; 6.4–8.6)	15 (5.9; 3.0–8.8)	140 (7.8; 6.6–9.0)	53 (7.7; 5.7–9.7)
1–2 times a month	85 (2.0; 1.6–2.4)	31 (3.1; 2.0–4.2)	39 (1.7; 1.2–2.3)	9 (3.5; 1.3–5.8)	40 (2.2; 1.6–2.9)	22 (3.2; 1.9–4.5)
1–2 times a week	380 (9.0; 8.1–9.9)	93 (9.4; 7.6–11.2)	148 (6.6; 5.6–7.7)	10 (3.9; 1.5–6.3)	214 (11.9; 10.4–13.4)	80 (11.6; 9.2–14.0)
3–4 times a week	246 (5.8; 5.1–6.5)	69 (6.9; 5.4–8.5)	101 (4.5; 3.7–5.4)	14 (5.5; 2.7–8.3)	135 (7.5; 6.3–8.7)	52 (7.5; 5.6–9.5)
At least 5 times a week	174 (4.1; 3.5–4.7)	44 (4.4; 3.2–5.7)	66 (3.0; 2.3–3.7)	5 (2.0; 0.3–3.7)	95 (5.3; 4.3–6.3)	27 (3.9; 2.5–5.4)

Depression symptoms coded as ≥80th percentiles, no depression symptoms coded as <80th percentiles

Depressive symptoms: Overall, a significantly higher proportion of the girls (2485/690, 27.8%; 95% CI: 26.0–29.5) had depressive symptoms compared with the boys (2485/255, 10.3%; 95% CI: 9.1–11.5). Overall, and in gender subgroups, those with depressive symptoms reported significantly poorer family economy, more smoking and consumption of alcohol, compared with the rest of the study population.

Overall, among students who participated in physical activity less than 3 times per week, a higher proportion reported depressive symptoms compared with students fulfilling more than 3 times per week. Additionally, adolescents who exercised or competed in a sports club less than once a week reported more depressive symptoms compared to those who reported 1 or more times per week.

There were gender differences. Of those who reported being physically active 1–2 times per week in a sports club, a smaller proportion of the girls (7.5% compare to 10.5%) and a higher proportion of boys (11.8% compare to 10.6%) reported depressive symptoms. Further, of those who went to the gym 1–2 times a week, a smaller proportion of the boys (12.2% compared to 18.1%) and a higher proportion of girls (21% compared to 18.3%) reported depressive symptoms.

Table 3 presents descriptive characteristics for adolescents participating in physical activity taking place in different contexts in adolescents aged 15–16 years in 2017.

**Table 3:** Youth data surveys: baseline characterstic of physical activity taking place in different contexts in adolescents aged 15–16 years in 2017

Variables	Sportsclub	Gym	Other organized PA (dance, martial arts etc.)	Independently (running, swimming, cycling etc)	No regular exercise
Depression symptoms					
No	589 (88.4%)	480 (78.8%)	141 (81.5%)	388 (78.1%)	623 (75.2%)
Yes	77 (11.6%)	129 (21.2%)	32 (18.5%)	109 (21.9%)	205 (24.8%)
Gender					
Male	341 (53.5%)	304 (52.9%)	50 (29.8%)	230 (47.9%)	400 (50.1%)
Female	296 (46.5%)	271 (47.1%)	118 (70.2%)	250 (52.1%)	398 (49.9%)
Parents higher education					
Both parents	426 (70.8%)	314 (58.8%)	114 (71.7%)	230 (52.5%)	347 (51.2%)
One of the parents	99 (16.4%)	126 (23.6%)	30 (18.9%)	108 (24.7%)	163 (24.0%)
None of the parents	77 (12.8%)	94 (17.6%)	15 (9.4%)	100 (22.8%)	168 (24.8%)
Family economy					
Good economy	557 (85.0%)	476 (79.3%)	133 (78.2%)	368 (75.3%)	559 (68.8%)
Neither good nor bad economy	80 (12.2%)	91 (15.2%)	28 (16.5%)	88 (18.0%)	176 (21.6%)
Poor economy	18 (2.7%)	33 (5.5%)	9 (5.3%)	33 (6.7%)	78 (9.6%)
Smoking					
No	627 (94.0%)	504 (83.0%)	154 (89.5%)	464 (93.2%)	735 (88.7%)
Yes	40 (6.0%)	103 (17.0%)	18 (10.5%)	34 (6.8%)	94 (11.3%)
Alcohol consuming					
< 1 time a month	608 (90.9%)	480 (78.8%)	156 (90.2%)	457 (91.8%)	739 (89.3%)
> 1–3 times a month	61 (9.1%)	129 (21.2%)	17 (9.8%)	41 (8.2%)	89 (10.7%)

Depression symptoms coded as ≥80th percentiles, no depression symptoms coded as <80th percentiles

Only one physical activity context (nr 1, 9, 13, 15 or none 16). *PA* Physical activity

Overall, 11.6% of the adolescents who participated in a sports club reported depressive symptoms. In other PA activity contexts the proportion of those with depressive symptoms varied between 18.5 - 21.9%. Approximately 71% of the adolescents attending a sports club had both parents with higher education, which was similar to those participating in other organized PA activities. In other PA activities the proportion varied between 52.5 and 58.8%. The majority of the adolescents who participated in other organized physical activities were girls (70.2%). Among adolescents who went to the gym 17.0% smoked and 21.2% drank alcohol more than 1–3 times a month. In other PA activity contexts the proportion varied between 8.2 and 9.8% for alcohol consumption and 6.0–10.5% for smoking.

Among adolescents not exercising regularly, 24.8% reported depressive symptoms compared with 11.6% among adolescents exercising in a sports club regularly.

Table 4 shows the results of binary logistic regression with the dichotomised depressive symptoms scale as the dependent variable.

**Table 4:** Binominal logistic regression of depressive symptoms in relation to different physical activity contexts

Variables	Model A		Model B		Model C		Model D		Model E		Model F	
Physical activity context	B	OR (95% CI)	B	OR (95% CI)	B	OR (95% CI)	B	OR (95% CI)	B	OR (95% CI)	B	OR (95% CI)
No regular exercise	0	1 (ref)	0	1 (ref)	0	1 (ref)	0	1 (ref)	0	1 (ref)	0	1 (ref)
1. Sportsclub	−0.923	0.40 (0.30–0.53)	− 0.911	0.40 (0.30–0.54)	− 0.941	0.39 (0.28–0.54)	− 0.841	0.43 (0.31–0.60)	− 0.797	0.45 (0.33–0.63)	− 0.781	0.46 (0.33–0.64)
2. Sportsclub and gym	− 0.918	0.40 (0.28–0.56)	− 0.733	0.48 (0.34–0.69)	− 0.806	0.45 (0.30–0.66)	−0.717	0.49 (0.33–0.72)	− 0.738	0.48 (0.32–0.71)	− 0.756	0.47 (0.32–0.70)
3. Sportsclub and other organized PA	−0.421	0.66 (0.42–1.02)	− 0.701	0.50 (0.31–0.80)	− 0.676	0.51 (0.31–0.83)	− 0.635	0.53 (0.32–0.87)	− 0.589	0.56 (0.34–0.92)	− 0.561	0.57 (0.35–0.94)
4. Sportsclub and independently	− 0.649	0.52 (0.38–0.72)	− 0.612	0.54 (0.39–0.76)	− 0.563	0.57 (0.40–0.81)	− 0.518	0.60 (0.42–0.85)	− 0.453	0.64 (0.44–0.91)	− 0.435	0.65(0.45–0.93)
5. Sportsclub, gym and other organized PA	−0.056	0.95 (0.51–1.76)	0.018	1.02 (0.53–1.94)	0.180	1.20 (0.61–2.34)	0.176	1.19 (0.60–2.37)	0.104	1.11 (0.55–2.25)	0.072	1.07 (0.53–2.20)
6. Sportsclub, gym and independently	−0.552	0.58 (0.41–0.81)	− 0.317	0.73 (0.51–1.04)	− 0.313	0.73 (0.50–1.06)	−0.247	0.78 (0.54–1.40)	− 0.196	0.82 (0.56–1.20)	−0.202	0.82 (0.56–1.20)
7. Sportsclub, independently and other organized PA	−0.264	0.77 (0.48–1.23)	−0.394	0.67 (0.41–1.12)	− 0.345	0.71 (0.42–1.20)	−0.223	0.80 (0.47–1.36)	− 0.143	0.87 (0.51–1.48)	−0.133	0.88 (0.51–1.50)
8. Sportsclub, gym, independently and other organized PA	−0.335	0.72 (0.46–1.11)	−0.490	0.61 (0.37–1.02)	− 0.514	0.60 (0.35–1.02)	−0.437	0.65 (0.38–1.11)	− 0.387	0.68 (0.40–1.17)	−0.385	0.68 (0.40–1.17)
9. Gym	−0.202	0.82 (0.64–1.05)	−0.157	0.85 (0.66–1.11)	− 0.209	0.81 (0.61–1.08)	− 0.158	0.85 (0.64–1.15)	− 0.183	0.83 (0.62–1.12)	−0.209	0.81 (0.60–1.10)
10. Gym and other organized PA	0.141	1.15 (0.71–1.87)	−0.016	0.98 (0.59–1.64)	0.014	1.01 (0.59–1.74)	0.086	1.09 (0.63–1.88)	0.116	1.12 (0.65–1.95)	0.078	1.08 (0.62–1.88)
11. Gym and independently	−0.117	0.89 (0.66–1.20)	−0.225	0.80 (0.58–1.10)	− 0.210	0.81 (0.58–1.13)	−0.186	0.83 (0.59–1.16)	− 0.236	0.79 (0.56–1.11)	−0.253	0.78 (0.55–1.10)
12. Gym, independently and other organized PA	−0.044	0.96 (0.54–1.69)	−0.357	0.70 (0.38–1.29)	− 0.551	0.58 (0.29–1.30)	−0.442	0.64 (0.33–1.26)	− 0.531	0.59 (0.30–1.17)	−0.578	0.56 (0.28–1.12)
13. Other organized PA (dance, martial arts etc)	−0.371	0.70 (0.46–1.04)	−0.618	0.54 (0.35–0.83)	− 0.658	0.52 (0.33–0.82)	−0.610	0.54 (0.34–0.86)	− 0.655	0.52 (0.33–0.83)	−0.638	0.53 (0.33–0.85)
14. Independently and other organized PA	−0.338	0.71 (0.46–1.12)	−0.724	0.49 (0.30–0.78)	− 0.816	0.44 (0.27–0.73)	−0.787	0.46 (0.28–0.75)	− 0.770	0.46 (0.28–0.78)	−0.752	0.47 (0.28–0.79)
15. independently (running, swimming, cycling etc)	−0.158	0.85 (0.66–1.11)	− 0.191	0.83 (0.63–1.09)	−0.231	0.79 (0.59–1.07)	− 0.186	0.83 (0.61–1.13)	−0.143	0.87 (0.64–1.18)	− 0.127	0.88 (0.65–1.20)
Gender												
Boy			0	1 (ref)	0	1 (ref)	0	1 (ref)	0	1 (ref)	0	1 (ref)
Girl			1.231	3.42 (2.91–4.04)	1.270	3.56 (2.99–4.25)	1.251	3.50 (2.92–4.18)	1.295	3.65 (3.05–4.37)	1.303	3.68 (3.07–4.42)
Parents education												
Parents_education, both					0	1 (ref)	0	1 (ref)	0	1 (ref)	0	1 (ref)
Parents_education, one of the parents					0.168	1.18 (0.97–1.45)	0.117	1.12 (0.92–1.38)	0.114	1.12 (0.91–1.38)	0.134	1.14 (0.93–1.41)
Parents_education, none of the parents					0.077	1.08 (0.86–1.36)	−0.044	0.96 (0.76–1.21)	−0.034	0.97 (0.76–1.23)	−0.002	1.00 (0.79–1.27)
Family economy												
Fam_economy, good economy							0	1 (ref)	0	1 (ref)	0	1 (ref)
Fam_economy, neither good nor bad							0.345	1.41 (1.14–1.75)	0.333	1.39 (1.12–1.73)	0.350	1.42 (1.14–1.77)
Fam_economy, poor economy							0.861	2.37 (1.70–3.29)	0.819	2.27 (1.62–3.17)	0.836	2.31 (1.65–3.23)
Do you smoke?												
No									0	1 (ref)	0	1 (ref)
Yes									0.829	2.29 (1.79–2.94)	0.539	1.72 (1.28–2.31)
Alcohol consuming												
< 1 time a month											0	1 (ref)
> 1–3 times a month											0.480	1.62 (1.24–2.10)

The dependent variable consisted of two categories coded asdepression symptoms (> 80 percentiles) and no depression symptoms (< 80 percentiles). The reference category was no depression symptoms

Model A: PA context and symptoms of depression, Model B: PA context, symptoms of depression and gender, Model C: PA context, symptoms of depression, gender and parents’ education, Model D: PA context, symptoms of depression, gender, parents’ education and family economy, Model E: PA context, symptoms of depression, gender, parents’ education and family economy and smoking, Model F: PA context, symptoms of depression, gender, parents’ education, family economy, smoking and alcohol consumption

*OR* Odds ratio, *95%* Confidence interval

In the crude model only including the composite physical activity variable, the odds of having symptoms of depression were lower for those who were physically active in a sports club (OR: 0.40, 95% CI: 0.30–0.53), in a sports club and gym (OR: 0.40, 95% CI: 0.28–0.56), in a sports club and exercising or keeping fit independently (OR: 0.52, 95% CI: 0.38–0.72) and in a sports club, gym and exercising or keeping fit independently (OR: 0.58, 95% CI: 0.41–0.81), compared to those who did not do regular exercise. Adjustment for gender did not change the OR for sports club, however, sports club together with other organized PA (OR: 0.50), other organized PA (OR: 0.54) and exercising or keeping fit independently together with other organized PA (OR: 0.49) became significant. Furthermore, the odds ratios for sports club together with gym and independent PA became non-significant. After adjustment for other independent variables, only small changes in the OR were observed.

Also in the additional analysis, not reported in tables, including adolescents from grades 8 and 9, the odds in the crude model of having symptoms of depression were lower for those who were physically active in a sports club (grade 8: OR: 0.47, 95% CI: 0.36–0.63, grade 9: OR: 0.44, 95% CI: 0.34–0.58), compared to those who did not do regular exercise. After adjustment for gender, there was only a small change in OR for grade 8 (OR: 0.5) and grade 9 (OR: 0.45), however, organized PA (grade 8: OR: 0.64, grade 9: OR: 0.65) became significant. After adjustment for other confounders, only sports club (grade 8: OR: 0.63, grade 9: OR: 0.49) became significant.

## Discussion

This article contributes to the field by exploring how the physical activity context relates to symptoms of depression. The main finding from our study is that the strength of the association between physical activity and symptoms of depression depends on the physical activity contexts. Participation in physical activity in a sports club setting was related to fewer depressive symptoms. While our findings are consistent with previous studies reporting increased benefits of participating in a sports club, e.g. lower depressive symptoms [13, 28, 29], our study expands previous research by relying on the concept of physical activity modalities, which has been operationalized and measured in terms of 16 physical activity categories. The explanation for the reported patterns of association remain tentative. A recent study reported that the benefit of team sports on depressive symptoms and positive mental health, was not explained by an increase in PA volume [28]. This is in line with a longitudinal study, which reported no association between volume of physical activity and the development of depressive symptoms [30]. Some studies have suggested a psychological effect of exercise as a possible contributing factor to the strong association between sports participation and mental health. Other studies have emphasized belonging to an extended group and increasing the number of friends adolescents have, as contributing factors that could explain some of the associations [10] between physical activity in sports clubs and lower symptoms of depression. When children and adolescents feel autonomous, socially connected to others, and competent they are more likely to enjoy the activity [31]. The association between sports club and fewer depressive symptoms can be explained by the social nature of team sport [32], alongside the positive involvement of peers and adults.

The results for grade 10 students indicate that it is not the background factors (such as gender, parents’ education, family economy, smoking or alcohol consumption) that explain the relationship between PA and symptoms of depression. However, in grade 8, it seems that background factors are more important compared to grade 10 as well as grade 9. This is consistent with a study among Canadian adolescents aged 12–13 years, which reported the association between physical activity and symptoms of depression to be non-significant when covariates were controlled for [13]. The adolescents’ transition from grade 8 to 10 is an important formative period of their lives in which friends and social settings beyond the family become more important.

After controlling for gender in this study, other organized physical activities became significant. Dance and martial arts were mentioned in the questionnaire as examples of other organized physical activities. This might reflect the fact that 70% of the adolescents who participated in other organized activities were girls. These patterns were also observed for grades 8 and 9, although these associations became non-significant after controlling for socioeconomic and other factors.

The finding in our study that adolescents who exercise or keep fit independently (running, swimming, cycling etc.) had a higher prevalence of symptoms of depression compared to those participating in a sports club is consistent with previous research [16, 17]. The direction of the association, however, cannot be determined from cross-sectional studies.

The methodological quality of many studies on adolescents has been questioned, particularly in relation to measurements of mental health and physical activity in different studies and the neglect of factors such as social interactions and psychological climate [5]. We cannot rule out that the associations between physical activity and mental health may, at least to some extent have been distorted because of weaknesses in the measurement of physical activity.

Given that there is a decrease in organised sport participation during adolescence [20], selection processes affecting the outcomes may occur because drop-out of less healthy students, e.g. in sports club activities. However, this hypothesis was not confirmed in the current study showing relatively similar patterns and associations across all three grades.

## Conclusions

Participation in a sports club was associated with significantly lower odds of depression which indicates that it is important to consider PA in different contexts, not only PA frequency and PA volume when investigating the association between adolescents’ PA and mental health. Further research is needed to explore these associations.

## Strength and limitations

The data used in this study were collected in 2017 and provide an up-to-date description of key aspects of young people’s lives. This is a cross sectional study, which precludes inferences about causal relationships. Depressive symptoms may not only work as an outcome, but may also act as an exposure that is hampering physical activity or giving rise to preferences for particular forms of physical activity (for example, a preference for independent activities rather than social). Self-reported measures of physical activity were used, which might lead to misclassification or measurement error. We only have the participation rate for each secondary school as a whole (grade 8, 9 and 10 combined). Also, although the outcome measure works reasonable well psychometrically, there are some measurement deficiencies. In particular, the DIF may have some impact on the results although the major pattern remains unaffected. Although there are ways to resolve DIF, in the present study we retained the original outcome variable. While resolving the DIF item(s) would have increased the fit of the data to the Rasch model, we could not rule out a negative effect on the validity of the variable because we do not know whether or not the cause of the DIF is relevant to the content of the variable.

## Abbreviations

KoRus: Regional centres for drug rehabilitation
NOVA: Norwegian Social Research
OR: Odds ratio
PA: Physical activity
